# Size-dependent effects of the intestinal microbiota in juvenile Chinese alligators: implications for species protection

**DOI:** 10.1186/s12983-025-00572-4

**Published:** 2025-07-29

**Authors:** Wengang Li, Jingru Liu, Lulu Cui, Ke Sun, Yulin Gao, Qin Wang, Yongkang Zhou, Lan Mei, Pingsi Yi, XiaoBing Wu, ZhenPeng Yu, Tao Pan

**Affiliations:** 1 College of Life Sciences, Anhui Normal University, Wuhu, 241000 Anhui China; 2 The Anhui Provincial Key Laboratory of Biodiversity Conservation and Ecological Security in the Yangtze River Basin, Wuhu, 241000 Anhui China; 3 National Long-Term Scientific Research Base for Chinese Alligator Artificial Breeding and Protection in Anhui, Anhui Research Center for Chinese Alligator Reproduction, Xuancheng, 242034 Anhui China; 4 School of Health Science and Engineering, Ma’anshan University, Ma’anshan, 243000 Anhui China

**Keywords:** Weight, Microbiota, Structure, Function

## Abstract

Increasing the quality of offspring to optimize population reproductive efficiency represents a viable approach for increasing population size. The population of Chinese alligators has a growing age structure, but the high mortality rate of juveniles is a serious problem that needs to be solved. We investigated the relationship between the weight of juvenile Chinese alligators and the microbiota to improve the survival rate and provide better protection for Chinese alligators. The results revealed significant differences in body weight among the selected individuals. Among the 35 most abundant microbial genera in the different weight groups, four genera (*Stenoxybacter*, *Gracilibacteria*, *Absconditabacteriales_*(*SR1)* and *Saccharimonadales*) were significantly positively correlated with weight. These genera can help the host shape the anaerobic environment of the intestine, degrade organic acids and proteins, and promote the production of growth-promoting factors. This study provided valuable insights into the relationship between the microbiota and weight, along with theoretical guidance for improving the survival rate of juvenile Chinese alligators.

**Supplementary Information:**

The online version contains supplementary material available at 10.1186/s12983-025-00572-4.

## Introduction

The Chinese alligator is an endangered reptile [6] that has a K-selection reproductive strategy characterized by slow development, large adults, a small number of offspring but a large body size, low reproductive energy allocation, and a long generation cycle. Owing to the low growth rate of the population, recovering the population is difficult. The artificially bred population has formed a stable age structure. However, the high mortality rate of juvenile Chinese alligators is one of the difficulties in artificial breeding. For example, in 2020, 836 individuals hatched at Dajiang Farm in August 2019, and between March and April, a total of 403 juvenile Chinese alligators died, with a mortality rate of 48.21% (internal data from the farm). To further increase the population of Chinese alligators, increasing the number of high-quality of offspring in the population to optimize the reproductive efficiency of the population represents a viable approach. Prey can resist predators through habitat selection, camouflage, and growth patterns [4, 24]. Newborn animals do not have fully developed means of resisting natural enemies. Along with the habitat selected by the parents, the size of the individual also helps the individual survive. For example, the larvae of many crustaceans have antennae, dorsal spines, and rostral spines that spread out when attacked, making the larvae larger and making it harder for predators to catch them [20]. However, this defense mechanism is not present in many reptiles, thus, a rapid increase in their body size can also help them survive infancy. Studies have shown that a turtle’s allosteric growth pattern may allow the hatchlings to pass the vulnerable stage of infancy more quickly and protect them from some predators [23]. Small size differences among juvenile side-blotched lizards can also affect individual competitive advantages and survival rates [9]. Additionally, according to our previous observations, compared to small juvenile alligators of the same age, larger individuals move farther, eat more times, and are more active. Therefore, under breeding conditions, larger individuals may gain a greater survival advantage. Moreover, the size growth rate of reptiles is an important indicator of an individual’s nutritional intake and health status [21]. Studies have assessed the habitat environment after returning the growth rate and ecological and nutritional intake of alligators in the United States [2]. Therefore, increasing the weight of juvenile alligators through human intervention is important for protecting the Chinese alligator population.

The animal gut microbiota not only protects the host against adverse changes in the external environment but also plays an important role in preventing the development of neurodevelopmental disorders, cancer, and other diseases [15, 26], The gut microbiota is closely related to the evolution of the host, which may have influenced the evolution of the host species and the evolutionary trajectory of the community for millions of years [10, 25]. The ability of crocodiles to adapt to their environment and their anticancer effects is most likely related to their gut microbiota, where 70–80% of immune cells may be present, and their complex relationship with the gut microbiota [32], Additionally, the gut microbiota of crocodilians produce molecules that may contribute to their cold tolerance [26]. Concerning immunity, gut microbiota produce metabolites that act against the immune system and regulate immune responses, which play a central role in cell signaling, inflammation, and interactions with immune cells [3]. Intestinal microbiota can promote the production of tryptophan and short-chain fatty acids in the intestine to enhance the production of antimicrobial peptides in the body [16]. Various immune cells, such as macrophages and dendritic cells, are closely related to the gut microbiota and its metabolites and play key roles in maintaining intestinal homeostasis and pathogen recognition [12]. The isolation and culture of intestinal microbiota of *Cuora amboinensis* revealed that *Pseudomonas aeruginosa* had a strong inhibitory effect on gram-positive and gram-negative bacteria [1]. The vertebrate gut microbiota is dominated by Bacteroidetes and Firmicutes, which affect the physiological metabolism and immune system of the host [18]. The core microbial community of reptiles is composed of Proteobacteria, Firmicutes, and Bacteroidetes. Additionally, reptiles have a gut microbiota that is more similar to birds than mammals [7]. Studies on *Crocodylus porosus* found the gut microbiota to be dominated by Firmicutes, including mainly *Clostridium*, suggesting that the gut microbiota of *C. porosus* is different from that of mammals, fish, and other reptiles [32]. The abundance of Fusobacteria in alligators makes their gut microbiota different from those of *C. porosus* [13].

To summarize, in the infancy stages of reptiles, larger individuals have a higher survival rate, and the intestinal microbiota can promote individual growth. This study used juvenile Chinese alligators of different body sizes to investigate the relationships between the body size and the microbiota. This study was conducted to identify probiotics to improve the survival rate of juvenile Chinese alligators and provide better protection.

## Materials and methods

### Grouping and sample collection

**Fig. 1:**
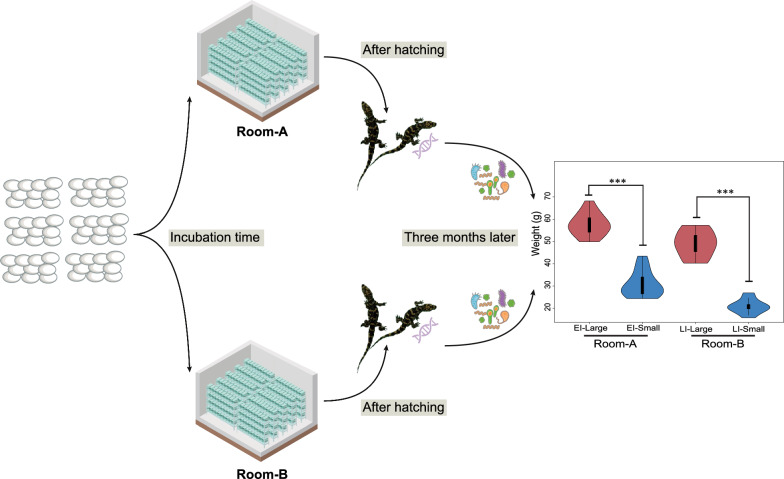
Schematic diagram of grouping and sampling

In 2021, 1000 Chinese alligators were incubated at Dajiang Farm in Wuhu. After each nest of eggs is laid, it is cleaned by staff and placed in a unified incubation area for incubation. The nesting materials used for incubation were disinfected uniformly before use. The temperature and humidity in the incubation area were held constant. Based on the different incubation times, early incubating juvenile Chinese alligators were placed in Room_A, and later incubating individuals were placed in Room_B when the number of Room_A individuals reached saturation (Room_A had early incubating (EI) individuals and Room_B had later incubating (LI)individuals). The maximum capacity of rooms A and B is 500 juvenile Chinese alligators. These numbers serve as the overall sample size, and subsequent grouping and sampling are based on these samples. There were 50 feeding troughs in Room_A and Room_B, with 10 juvenile alligators in each feeding trough, which were fed once a day. The same amount of food was provided each time, and the water was changed every three days. The animals were maintained in a standardized breeding farm, and the temperature (32 ℃), humidity (70%), and cleaning frequency (once every three days) of each room were consistent. The juvenile alligators in Room_A and Room_B were raised in the same way for three months. After each individual reached three months of age, the physiological data (body length i.e., length from snout to tail, and weight) of the animals were measured, and cloacal microbial samples were taken. According to the grouping, 15 samples were from EI_Large and EI_Small, 12 from LI_Large, and 12 from LI_Small, for 54 total samples (Fig. 1). Before sampling, the whole bodies of the small Chinese alligators were disinfected with an iodophor, the alligators were placed on the tray of the sterile operating table, and a sterile cotton swab was inserted into the cloaca 0.5–1 cm deep. The swab was rotated clockwise and carefully pulled out, placed in a sterile tube, and stored at − 80 °C.

### DNA extraction, PCR amplification, library construction and sequencing

The genomic DNA of the samples was extracted using the CTAB method [19], after which the purity and concentration of the extracted DNA were determined via agarose gel electrophoresis. Following the method described in previous studies, PCR amplification of the 16S rRNA gene sequence was performed by targeting the V3-V4 region using a set of forward and reverse primers. The forward and reverse priming sequences used in this experiment are as follows: Pro_341F (5’-ATCCTACGGGAGGCAGCA-3’) and Pro_806R (5’-GGACATACHVGGGTWTCTAAT-3’) [28]. PCR was performed in a total volume of 25 μL containing 2X KAPA HiFi HotStart Ready Mix (12.5 μL), 400 nM each primer, ddH_2_O (7.5 μL), and 3 μL of template DNA. PCR was performed in a Px2 Thermal Cycler (Thermo, USA) under the following conditions: initial denaturation at 95 °C for 30 s, followed by denaturation at 95 for 30 s, annealing at 55 °C for 30 s, and extension for 30 s at 72 °C for 28 cycles. Amplicons were identified by 30 min of electrophoresis on a 1.5% agarose gel at 110 V, following the manufacturer’s instructions. The Nextera XT DNA Library Preparation Kit (Illumina) was used to construct libraries from isolated DNA. After separation via agarose gel electrophoresis, PCR products of the desired size were purified from the substrate. The amplification products were sequenced on the MiSeq Illumina platform (Illumina, Inc., San Diego, CA, USA) using a paired method.

### Amplicon sequence variant (ASV) clustering and species notes

The sequence quality file was checked using DADA2 (divisive amplicon denoising algorithm 2) [5], the correct sequence was selected for filtering and pruning, and then it was modeled according to the error rate. Based on this model, the sample sequence was inferred. Clean reads were obtained after the chimeras were removed. Clean reads were annotated and classified using the SILVA132 SSUrRNA database to obtain complete sequence species information.

### Data analysis

The relative abundance of microorganisms in each sample was calculated based on the pure ASV table, and the subsequent analysis was based on the relative abundance data. A t-test was performed to examine differences in the relative abundance of microbiota between groups by calculating indices (ACE, Good’s Coverage, PD Whole tree, and Simpson). A histogram was used to show the 15 most abundant species of microbiota at the genus and phylum levels. The microbiota belonging to the top 35 in terms of horizontal relative abundance were clustered to draw heatmaps based on Euclidean distance. The Bray–Curtis dissimilarity matrix was calculated according to the relative abundance data and applied to the nonmetric multidimensional scale (NMDS). The microbial function was predicted using the PICRUSt2 software [8].

## Results

### Body size classification

**Fig. 2:**
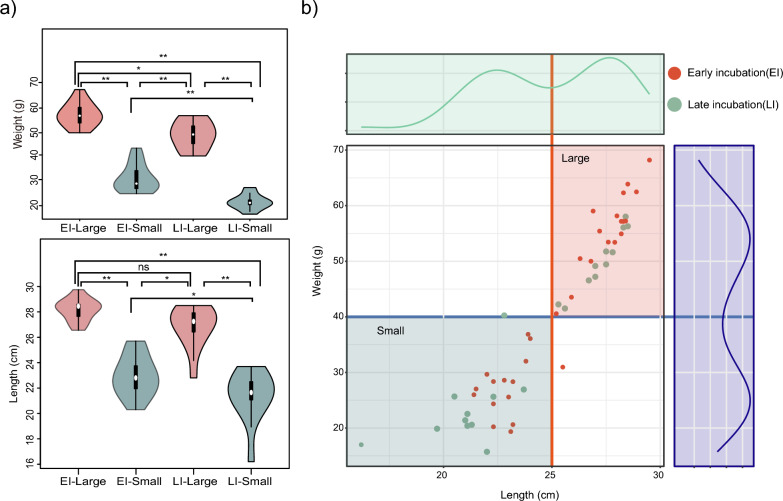
Overview of sample grouping and sequencing data. **a** Analysis of intergroup body size differences, via t-test for difference analysis (“*” represents “0.01 < *p* < 0.05”; “**” represents “*p* < 0.01”; “ns” represents “Not significant”). **b** Scatter plot of the body size distribution of juvenile Chinese alligators. The abscissa represents the body length, and the ordinate represents the weight. The curve on the right represents the probability density distribution of weights, and the upper curve represents the probability density distribution of body length. The red dots represent individuals who hatched first, and the green dots represent individuals who hatched later

We first performed a comparative analysis of body size among the samples, and the results revealed significant differences in body weight across the four groups. Compared to those in the other three groups, individuals in the EI_Large group had significantly greater body weights, whereas individuals in the LI_Small group had significantly lower body weights. In terms of body length, both EI_Large and LI_Small individuals were significantly longer than those in the other two groups, whereas individuals in the LI_Small group were notably shorter than those in the other three groups (Fig. 2a).

A scatter plot of body shape was generated, with body length plotted on the X-axis and body weight on the Y-axis. The plot also included probability density divisions for body length and weight. Red dots represent early-hatching individuals (EI), and green dots represent late-hatching individuals (LI). As shown in Fig. 2b, body length and body weight exhibited bimodal distributions, with two distinct peaks on the probability density curves. This indicated that the samples could be classified into two categories based on body size: large and small, as marked on the graph. Subsequent grouping was performed according to this classification.

### Data result availability

High-throughput sequencing was conducted on 54 samples (Fig. S1a). The cumulative curve indicated that the observed species value leveled off as the number of samples increased, suggesting that the sample size was appropriate and that the results were reliable. In the cloaca, the number of ASVs shared between large and small body size varied across the groups. In the first hatching group, seven ASVs were shared between the large and small body size. For the large body size, six ASVs were unique, whereas 19 ASVs were unique to the small body size. In the second hatching group, 28 ASVs were shared between the large and small body types, with seven ASVs unique to the large body size and four unique to the small body size (Fig S1b).

### Differences in microbial structure and composition during incubation

**Fig. 3:**
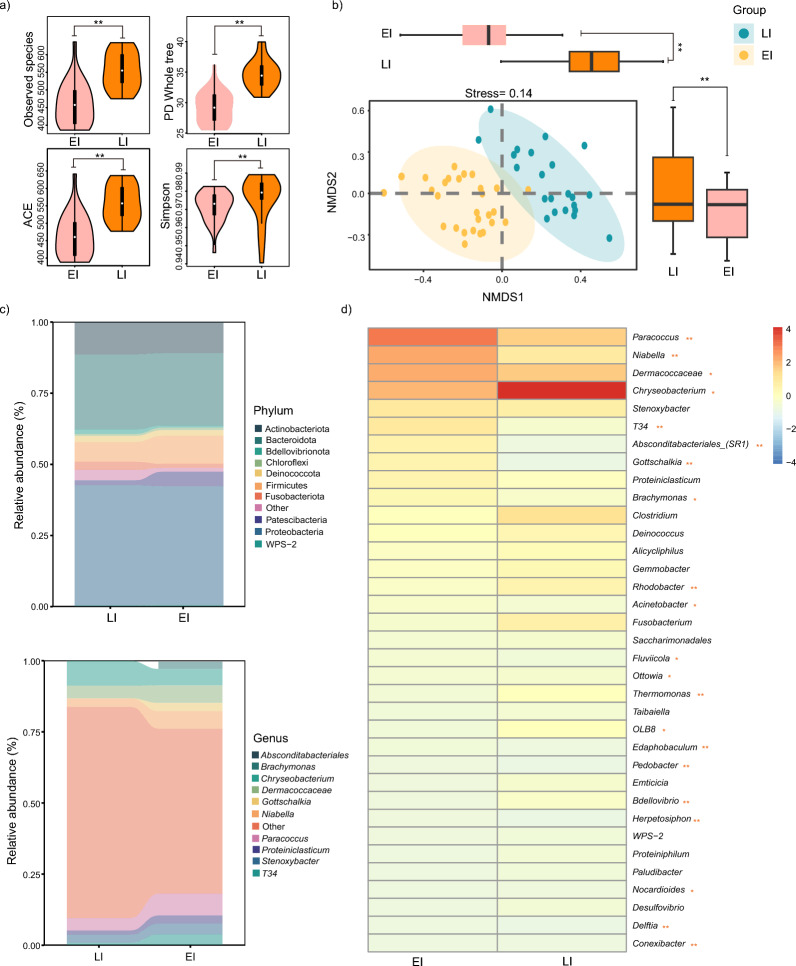
Analysis of microbial structure and composition differences over time. **a** Comparison of microbial alpha diversity between the first hatching group and the second hatching group. **b** Comparison between the first-hatching and second-hatching groups. Differences reduce the relative abundance of microorganisms to two dimensions for clustering, and the differences between the two groups in each dimension are compared (t-test). **c** Statistics of the top 10 microbial phyla and genera at the hatching group and hatching group levels. The horizontal axis represents the grouping, and the vertical axis represents the relative abundance of microorganisms. Each color represents the phylum and genus levels of the microorganisms. d) The top 35 statistics of the microbial genera at different incubation times indicate the relative abundance. “**” represents *p* < 0.01 (t-test), and “ns” represents *p* > 0.05 (t-test)

To analyze the structural differences in the microbiota of juvenile alligators from different hatching groups, we assessed four α diversity indices, including observed species, ACE, PD_whole tree, and Simpson. The results revealed significant differences in all four indices, indicating that individuals incubated for different durations presented notable differences in microbiome richness, evenness, and abundance (Fig. 3a). To further investigate these differences, dimensionality reduction, and cluster analysis were performed based on the relative abundance of microbiota in each sample. The results (Fig. 3b) showed a clear separation between the two groups of samples with different incubation times, with highly significant observed in both dimensions after dimensionality reduction. The samples could be clustered into two distinct groups, and the analysis was found to be credible (stress = 0.14). Next, we analyzed the top 10 phyla and genera in the two sample groups. The results revealed that both groups were composed of 10 phyla, including Actinobacteria, Bacteroidota, Bdellovibrionota, Chloroflexi, Deinococcota, Firmicutes, Fusobacteriota, Patescibacteria, Proteobacteria, and WPS-2. At the genus level, the predominant genera were *Absconditabacteriales_(SR1)*, *Brachymonas*, *Chryseobacterium*, *Dermacoccaceae*, *Gottschalkia*, *Niabella*, *Paracoccus*, *Proteini*, *Stenoxybacter*, and *T34* (Fig. 3c). To further assess the differences in genus composition, a heatmap was used to display the top 35 genera in the two groups and a statistical comparison was made. The results revealed that 21 of the top 35 genera differed significantly between individuals with different incubation times. The abundance of *Chryseobacterium* was significantly lower in the EI group than in the LI group, whereas the abundances of *Paracoccus* and *Niabella* were significantly greater in the EI group (Fig. 3d).

### Analysis of the microbiological composition and structure of juvenile Chinese alligators with body size differences

**Fig. 4:**
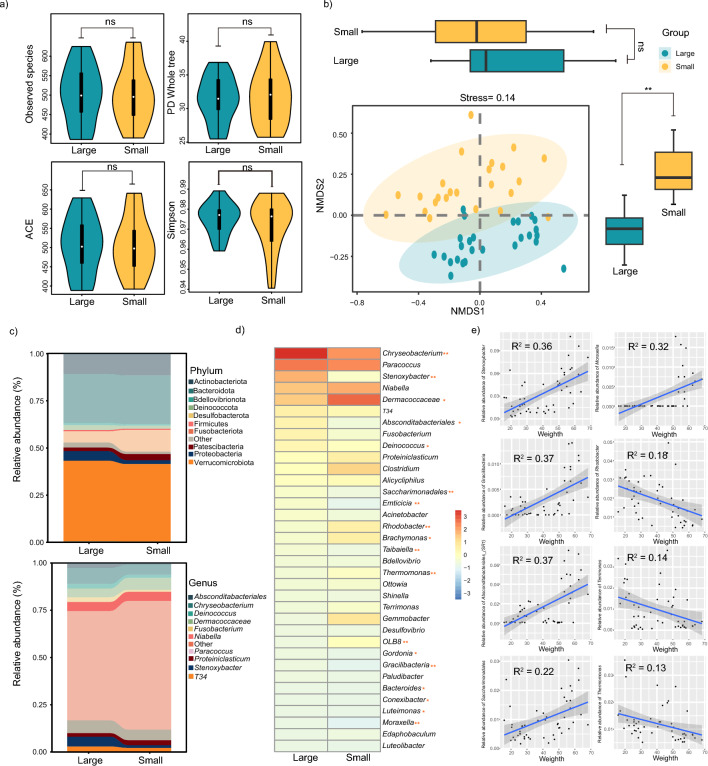
Analysis of microbial structure and composition differences under the background of body size differences. **a** Comparison of microbial alpha diversity between the large- and small-body groups; **b** Intergroup differences between the large- and small-body size groups. The relative abundance of microorganisms was reduced to two dimensions for clustering, and the differences between the two groups in each dimension were compared (t-test). **c** Statistics of the top 10 microbial phyla and genera in the large-scale and small-scale groups. The horizontal axis represents the grouping, and the vertical axis represents the relative abundance of microorganisms. Each color represents a microbial phylum and genus. d) The microbial genera associated with large-size and small-size groups are the top 35, and the color represents the relative abundance. “**” indicates *p* < 0.01 (t-test), and “ns” indicates *p* > 0.05 (t-test). Analysis of differences in microbial structure between large-sized and small-sized groups: microbial composition of large-sized groups and correlation analysis between body size and microorganisms

To determine whether there were differences in microbial composition and structure between juvenile Chinese alligators of different body sizes, we first performed statistical analysis on four α diversity indices: observed Species, PD whole tree, ACE, and Simpson indices. The results revealed no significant differences in these indices between the two groups (Fig. 4a). This suggests that no substantial differences were present in the abundance, richness, or evenness of cloacal microorganisms between the two body size groups. Next, dimensionality reduction was applied based on the relative abundance of microorganisms. After dimensionality reduction, the samples clustered into two distinct groups (Fig. 4b), with a highly significant difference observed in one factor (NMDS2), and the results were credible (stress = 0.14). To visualize the microbial composition, we plotted cumulative bar charts of the top 10 phyla and genera for both groups. The top 10 phyla identified were Actinobacteriota, Bacteroidota, Bdellovibrionota, Deinococcota, Desulfobacterota, Firmicutes, Fusobacteriota, Patescibacteria, Proteobacteria, and Verrucomicrobiota. The top 10 genera included *Absconditabacteriales_(SR1)*, *Chryseobacterium*, *Deinococcus*, *Dermacoccaceae*, *Fusobacterium*, *Niabella*, *Paracoccus*, *Proteiniclasticum*, *Stenoxybacter*, and *Gracilibacteria* (Fig. 4c).

To further evaluate the relationship between body size and microbial composition, we displayed the top 35 genera using a heatmap (Fig. 4d) and performed a t-test to assess differences between the groups. The analysis revealed significant differences in 18 of the top 35 genera between the two body size groups. Then, the correlations between these 18 genera and body weight were analyzed using linear regression analysis. The results (Fig. 4e) revealed that *Stenoxybacter*, *Gracilibacteria*, *Absconditabacteriales_(SR1*) and *Saccharimonadales* were strongly positively correlated with body weight, suggesting that they may be potential probiotics. In contrast, *Moraxella*, *Rhodobacter*, *Terrimonas*, and *Thermomonas* were significantly negatively correlated with body weight. The correlation between microorganisms and body length also exhibited a consistent pattern (Fig. S1).

### Microbial function analysis in individuals with body size differences

**Fig. 5:**
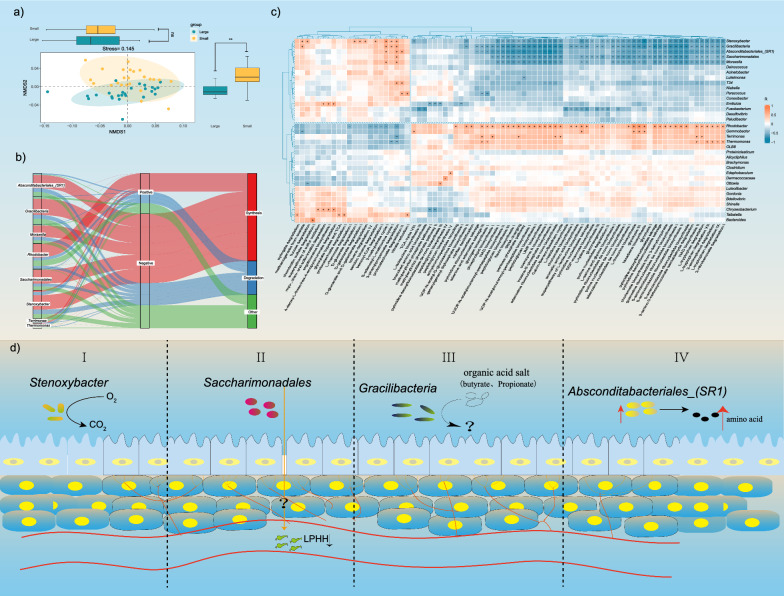
Analysis of of microbial functional differences with the background of body size differences. **a** Differences in microbial functional groups between the large-body and small-body groups. The relative abundance of microbial functions was reduced to two dimensions for clustering, and the differences between the two groups in each dimension (t-test) were compared. **b** Analysis of the correlation between two groups of differential microorganisms with different body size and the functions of the microbiota. The top 20 genera of microorganisms with significant differences in relative abundance between the large-sized and small-sized groups were selected, and Pearson correlation analysis was performed on the functional groups with significant differences. **c** Summary analysis of some microbial functions. **d** Schematic diagram of the effects of probiotics on the growth and development of the body. **e** Correlation between body weight and the relative abundance of microorganisms

To analyze functional differences between groups with varying body sizes, we first performed dimensionality reduction analysis based on functional abundance. The results revealed that after dimensionality reduction, the individuals from the two groups clustered into two distinct categories, with significant differences observed in the NMDS2 factor (Fig. 5a). These findings indicated that microbial functions differ between the large and small body size groups. A correlation analysis of eight microbial functions with body size revealed that functions positively correlated with body size were primarily involved in substance synthesis, whereas those negatively correlated with body size were predominantly related to substance degradation (Fig. 5b). To gain deeper insights into the differential functions, a correlation analysis was performed between the differential microorganisms and their associated functions and the results were illustrated in a heatmap. The heatmap (Fig. 5c) revealed 82 different functions. Among these functions, 22 microbial functions were positively correlated with body size, 15 of which were related to the synthesis and degradation of organic substances (e.g., eugenic acid and fucose), accounting for 68.2% of the microbial functions. In contrast, 36 microbial functions were negatively correlated with body size, 24 of which were related to the synthesis of organic substances (e.g., glycogen and adenosine ribonucleotide), accounting for 66.7% of the microbial functions..

## Discussion

Individuals with different body sizes have different compositions and structures of microorganisms, and their microbial functions also differ. *Stenoxybacter*, *Gracilibacteria*, *Absconditabacteriales_(SR1)*, *Saccharimonadales*, and *Moraxella* were positively correlated with body size. *Stenoxybacter* is widely found in the guts of insects such as termites [31]. It can use acetate as an energy source [31] and consume O_2_ in the gut. The decomposed acetate helps the body maintain the anaerobic environment in the gut [27, 30], and can promote its absorption and utilization. The relative abundance of *Absconditabacteriales_(SR1)* in the rumen of yaks was significantly greater than that in the untreated group after high-quality food was added, while the rumen methionine and lysine contents increased [11]. The relative abundance of *Gracilibacteria* in the intestinal microorganisms of sows fed a high-protein diet was significantly greater than that in the low-protein diet group, and a correlation with the blood metabolism group revealed that *Gracilibacteria* was positively correlated with vitamin C, histamine, diisolinolenic acid, naphthalene, and acetophenone but negatively correlated with cinnamaldehyde and 3-phenylpropionic acid [29]. The relative abundance of *Gracilibacteria* in the lamb rumen was negatively correlated with acetate, propionate, butyrate, and total volatile fatty acids [17]. In this study, *Gracilibacteria* was positively correlated with the degradation of 3-phenylpropionic acid and protocatechuate, as well as with weight gain. Acetate, propionate, and butyrate can reduce the average daily feed intake of mice, downregulate the mRNA expression of myosin heavy chain (MyHc) IIb, upregulate the mRNA expression of the lipase hormone-sensitive enzyme myHCiiA and carnitine palmitoacyltransferase-1A, and reduce fat accumulation in mice by regulating appetite and related genes [12]. In this study, the relative abundance of *Gracilibacteria* was positively correlated with the body weight of juvenile alligators and the degradation of various organic acids. We speculated that *Gracilibacteria* may promote the feeding of juvenile alligators by reducing the content of organic acids in the intestinal tract and increasing the accumulation of fat in juvenile alligators. The relative abundance of *Saccharimonadales* in the gut of mouse models of colon cancer was significantly greater than that in healthy controls, and the expression of *Saccharimonadales* and the phospholysine phosphistidine inorganic pyrophosphatase (LHPP) gene was negatively correlated with that of *Saccharimonadales* [11]. LHPP is a cancer suppressor, and its content is negatively correlated with the incidence of many types of cancer [33]. The main function of LHPP is to inhibit cell proliferation. In this study, the relative abundance of *Saccharimonadales* was positively correlated with body weight. By reducing the content of LHPP in the blood, *Saccharimonadales* can reduce the inhibition rate of cell proliferation in alligators, thus promoting individual growth. To summarize, *Stenoxybacter* can consume intestinal oxygen, help the body form an anaerobic environment in the intestine, and provide a suitable environment for the growth of other probiotics. The addition of *Absconditabacteriales_(SR1)* improved the utilization rate of proteins by the host. *Gracilibacteria* can promote the degradation of organic acids in the gut and help juvenile Chinese alligators accumulate fat. *Saccharimonadales* can reduce the content of LHPP in the blood, improve cell proliferation, and promote the growth and development of juvenile Chinese alligators. Therefore, the effect of probiotics on juvenile Chinese alligators does not involve a single strain; instead, different microorganisms interact with each other to change the content of active substances in the host body and promote the growth of juvenile Chinese alligators (Fig. 5d).

In this study, the microbial functions that were positively correlated with body size mainly included degradation of acids (eugenic acid, 3-phenylpropionic acid, S-adenosine-L-methionine, and D-galactoic acid), sugars (L-rhamnose, D-glucose, and fucose) and salts (protocatechuates, acetate, and nitrates). In human studies, 3-phenylpropionic acid (PPA) is a major metabolite produced by *Clostridium sporogenes* and may act as a unique chemical messenger to communicate with the host [14]. The amount of 3-phenylpropionic acid in the blood of diabetic patients is significantly greater than that in the blood of healthy people [22]. The degradation of organic salts and sugars in the gut can provide more absorbable secondary metabolites for the host. The microbial functions negatively associated with the body size of juvenile alligators are mostly related to the synthesis of bioactive substances, probably because the synthesis of substances by microorganisms requires the absorption of substrates from the host intestine. As microorganisms synthesize their active substances in cells, the amount of absorbable substrates in the intestines of juvenile Chinese alligators decreases. Therefore, this difference was reflected in the individual growth rates.

To summarize, the intestinal flora can change the morphology of intestinal contents, thereby promoting the growth and development of the host. This study revealed four potential probiotics. In the subsequent breeding and protection of juvenile Chinese alligators, this result can be included as one of the monitoring indicators for the health of juvenile Chinese alligators, The application of this indicator may improve the comprehensive ability of staff to manage the health of juvenile Chinese alligators.

## Supplementary Information

Additional file 1.

## Data Availability

All sequencing data generated in this study are available on PRJNA1000116 (https://www.ncbi.nlm.nih.gov/bioproject/PRJNA1000116/).
