# Supplementary material for: Size-dependent effects of the intestinal microbiota in juvenile Chinese alligators: implications for species protection
Source: Front Zool. 2025 Jul 29;22:15. doi: 10.1186/s12983-025-00572-4 (PMC12305974; doi:10.1186/s12983-025-00572-4)

**Supporting Documents**

**Fig. S1** a) Sample ASV cumulative curve. b) Statistics of the number of ASV numbers in different groups.
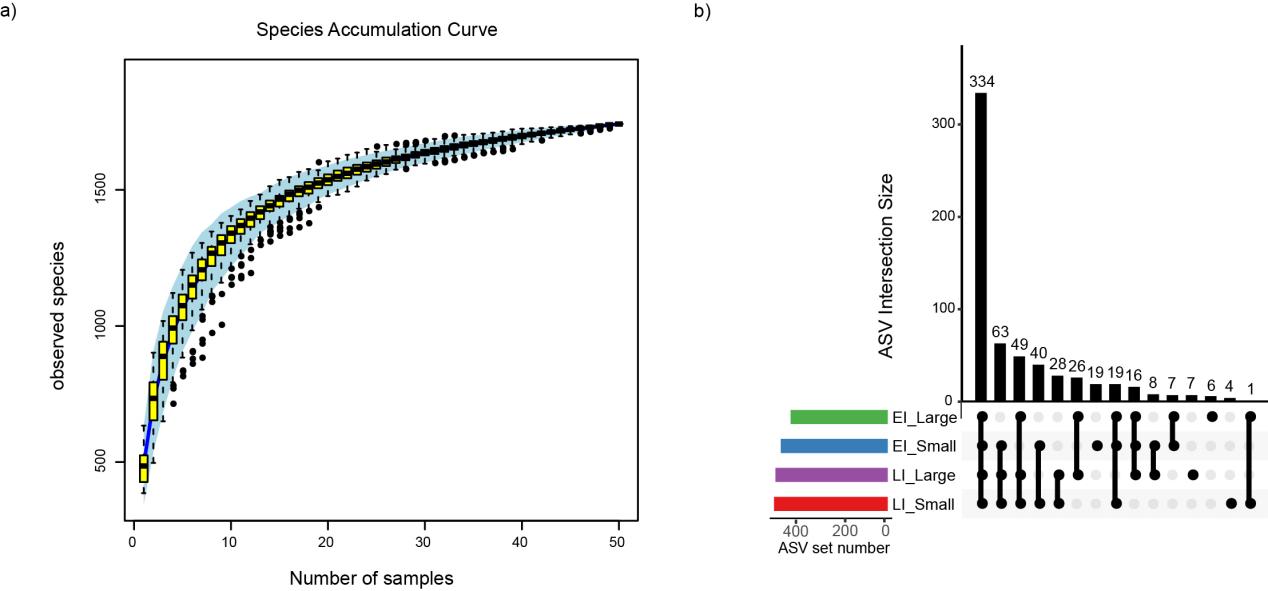


**Fig. S2** Correlations between body length and the relative abundance of microorganisms.


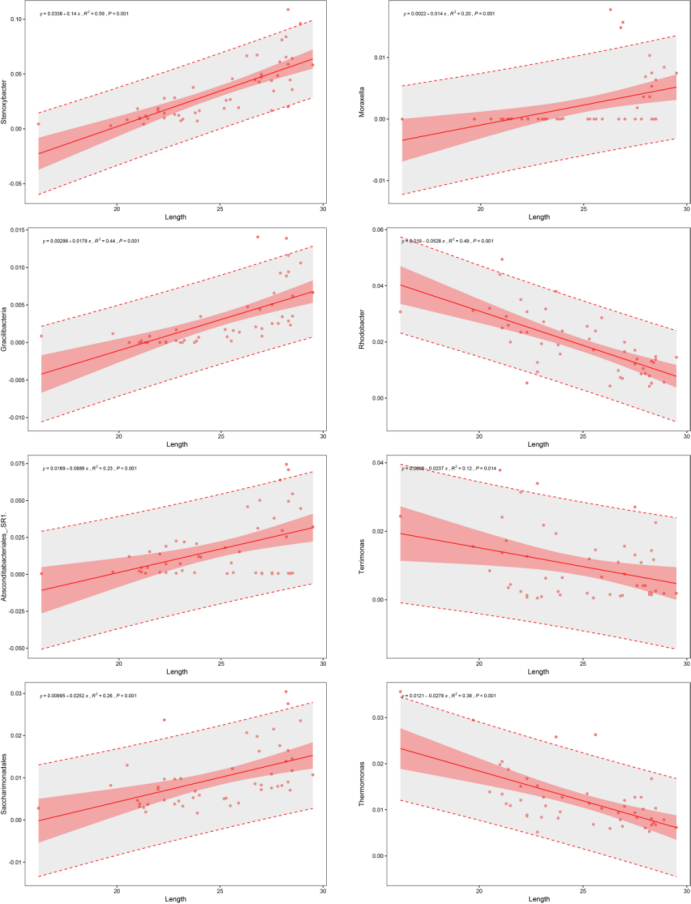

Supplement: Supplementary file 1 — Additional file 1. [file 12983_2025_572_MOESM1_ESM.docx]
